# Fecal Microbiota Analysis in Patients Going through a Depressive Episode during Treatment in a Psychiatric Hospital Setting

**DOI:** 10.3390/jcm8020164

**Published:** 2019-02-01

**Authors:** Paweł Liśkiewicz, Justyna Pełka-Wysiecka, Mariusz Kaczmarczyk, Igor Łoniewski, Michał Wroński, Agata Bąba-Kubiś, Karolina Skonieczna-Żydecka, Wojciech Marlicz, Błażej Misiak, Jerzy Samochowiec

**Affiliations:** 1 Department of Psychiatry, Pomeranian Medical University in Szczecin, Broniewskiego 26, 71-460 Szczecin, Poland; pjliskiewicz@gmail.com (P.L.); wysiecki@wp.pl (J.P.-W.); mwronski@pum.edu.pl (M.W.); agata0621@gmail.com (A.B.-K.); samoj@pum.edu.pl (J.S.); 2 Department of Clinical and Molecular Biochemistry, Pomeranian Medical University in Szczecin, Powstańców Wielkopolskich 72, 70-111 Szczecin, Poland; mariush@pum.edu.pl; 3 Department of Biochemistry and Human Nutrition, Pomeranian Medical University in Szczecin, Broniewskiego 24, 71-460 Szczecin, Poland; karzyd@pum.edu.pl; 4 Department of Gastroenterology, Pomeranian Medical University in Szczecin, Unii Lubelskiej 1, 71-252 Szczecin, Poland; marlicz@hotmail.com; 5 Department of Genetics, Wroclaw Medical University, Marcinkowskiego 1, 50-368 Wrocław, Poland; mblazej@interia.eu

**Keywords:** microbiota, hospital stay, escitalopram, depression

## Abstract

Rationale: There is a worldwide prevalence of generalized anxiety and major depressive disorders (MDD). Gut–brain axis dysfunction, antibacterial activity, and modulatory effects of antidepressants toward intestinal bacteria have been shown both in vitro and in vivo. Objectives: In this study, we aimed to investigate the effects of hospital stay, including escitalopram administration, on gut microbiota in patients with depressive episodes. Methods: After admission to the hospital and 7-days washout from all medications the composition of fecal microbiota samples was evaluated at baseline (W0) and after 6 weeks (W6), using 16S rRNA sequencing. The study was conducted on 17 inpatients (52.9% females), who followed the same daily hospital routine, including a standard diet and received 5–20 mg daily doses of escitalopram. Results: At the end of treatment (W6), no change was observed in the Chao1 index. However, Shannon (median (Q1–Q3): W0 2.78 (2.67–3.02) vs. W6 3.11 (2.80–3.30)), and inverse Simpson (median (Q1–Q3): W0 9.26 (7.26–13.76) vs. W6 12.13 (9.17–15.73)) indices increased significantly compared to baseline values (False Discovery Rate *p* (*q*) = 0.031 and *q* = 0.011, respectively). We also found that between-subject W0 Bray–Curtis dissimilarities were significantly higher than W0–W6 within-subject dissimilarities (median (Q1–Q3): 0.68 (0.56–0.77) vs. 0.38 (0.35–0.52), two sided Mann–Whitney test *p* < 0.00001. The within-subject dissimilarities did not depend on sex, age, BMI, illness duration and a daily dose of escitalopram. No significant differences between taxa levels, at the studied time points, were observed when adjusted for multiple hypotheses testing procedures. Conclusions: We conclude that a six-week treatment in a psychiatric hospital setting resulted in increased alpha biodiversity in fecal microbiota, however its causal relationship with patients’ mental health was not proved. We have also found that individual microbiome stability was not affected by hospitalization.

## 1. Introduction

Over 350 million people suffer from depression, constituting 4.4% of the world’s population. Depression affects people of all ages and social groups, with higher prevalence in women than in men (5.1% vs. 3.6%). Worldwide, it is the second leading cause of disability, just behind ischemic heart disease. Depression can lead to suicide, and it is estimated that close to 800,000 people die due to suicide every year; making it the second most common cause of death in people aged 15–29 [1]. In Poland, depression is prevalent in approximately 3.0% of the population, including both men and women at 1.9% and 4.0%, respectively [2].

Sadness, dejection, and malaise are not always symptoms connected to depressive syndrome diagnosis. In current clinical practice, depressive disorders are diagnosed according to the International Classification of Diseases, Tenth Revision (ICD-10) and The Diagnostic and Statistical Manual of Mental Disorders, 5th ed. (DSM-5) classification criteria. Depressive episodes may have a chronic, recurrent clinical course, or they may be isolated, discrete episodes that never recur. As the former is associated with increased co-morbidity and mortality [3], its course includes periods of relapse and remission.

Effective depression-related treatments include pharmacological and psychological interventions, which manage current affective episodes and prevent their future recurrence. In clinically justified cases, the treatment course is recommended to take place in a hospital setting.

Evidence indicates that besides monoamine deficiency [4], neurogenesis alterations [5], genes [6], and epigenetics [7], other mechanisms, such as psychological factors may play a role in this disease. The available data point to a close relationship between depression and inflammation. Elevated concentrations of proinflammatory cytokines, e.g., IL-6, IFN-γ, and IL-2 [8,9], could potentially affect the central nervous system (CNS) functions via direct and afferent pathways [10]. Consequently, factors influencing other inflammatory processes within an organism, such as alterations in the gut microbiota, and intestinal permeability, as well as subclinical inflammation within the gastrointestinal tract or other organs, may contribute to depression [11,12].

With the development of molecular techniques (pyrosequencing of 16s rRNA bacterial genes), evidence has emerged of intestinal microbiota creating active environments involved in many physiological processes [13], including those in CNS. The gut–brain axis (GBA)—involving various neural and biochemical mechanisms—can therefore be a starting point for developing new therapies for gastrointestinal and mental disorders. Unfortunately, disease-specific microbiome metrics have not been unified, however, the most often assessed include: Alpha diversity, number of taxonomic units within one ecosystem (e.g., number of genera in one fecal sample); and beta diversity, difference in the abundance of taxonomic units between ecosystems (e.g., the presence and counts of particular bacterial genera between two samples collected from two people). In this context, dysbiosis refers to alterations in the abundance of qualitative and/or functional microbiota differences [14].

In the adult digestive tract, the gut’s ecosystem is over-dominated by *Firmicutes*, followed by Bacteroidetes phyla [15]. In rodents manifesting depression-like behavior, and in clinical studies on patients diagnosed with depression, significant differences exist in the abundance of particular genera within *Bacteroidetes*, *Firmicutes*, *Proteobacteria*, and *Actinobacteria* phyla [16]. Changes in the composition of gut microbiota have been observed in depressive individuals, including changes in richness and diversity [17,18,19,20,21], bacterial abundance [19], and metabolic activity [22]. However, results vary for some genera, emphasizing that some confounders may be involved in the microbiota-based phenotype of depression in animal models [23].

Alterations in intestinal microbiota have been associated with the use of antidepressants. Since the introduction of the first antidepressant—a tuberculostatic drug (iproniazid) [24]—medications prescribed for sequential therapy have presented mainly with antimicrobial activity [25,26]. Antidepressants differ in mechanisms of their antibacterial activity; for example, they affect cellular respiration, whereby Monoamine Oxidase Inhibitors (MAOIs) can disturb bacterial cell-wall synthesis (Jena et al., 2014; Lei et al., 2000). Tricyclic antidepressants (TCAs) inhibit DNA gyrase activity, plasmid DNA replication, (Molnár, 1988), and the growth of *E. coli*, *Yersinia enterocolitica* [27], and *Giardia lamblia* [28,29]. Selective serotonin reuptake inhibitors (SSRIs) inhibit efflux pumps (Bohnert et al., 2011; Gjestad et al., 2015), exhibiting activity against *Staphylococcus* and *Enterococcus* [26,30,31], *Citrobacter* spp., *P. aeruginosa*, *K. pneumonia*, *M. morganii*, *Clostridium perfringens*, and *Clostridium difficile* [26,32]. Furthermore, commensal bacteria, including *E. coli* and *L. rhamnosus*, have shown sensitivity for these medications [33]. A few antibiotics, e.g., doxycycline and minocycline, exhibit antidepressant effects [34,35].

Escitalopram is an antidepressant of the SSRI class, mainly prescribed for the treatment of major depressive disorder (MDD) and generalized anxiety disorder (GAD). Its major mechanism of action involves increasing intrasynaptic levels of 5-hydroxytryptamine (5-HT), by blocking the neurotransmitter reuptake. The drug has antibacterial activity against *Staphylococcus aureus*, *Pseudomonas aeruginosa*, *Klebsiella pneumoniae*, *Proteus mirabilis*, and *Enterobacter cloacae* [36]. Using experimental animals, Cussotto et al. showed escitalopram’s antimicrobial action against *Escherichia coli* APC105, and its modulatory effect towards intestinal bacteria [37].

A literature-wide search did not retrieve any data on the resultant effects of treating MDD patients, with depressive episodes, on fecal microbiota composition. We, therefore, aimed to analyze microbiota of stool samples collected from a cohort of patients admitted to our clinic. This patient cohort comprised of individuals suffering from a depressive episode who also received escitalopram, to test the hypothesis that hospitalization (unified diet and environmental conditions) and treatment affect microbiota composition.

## 2. Material and Methods

### 2.1. Patients

**Figure 1:**
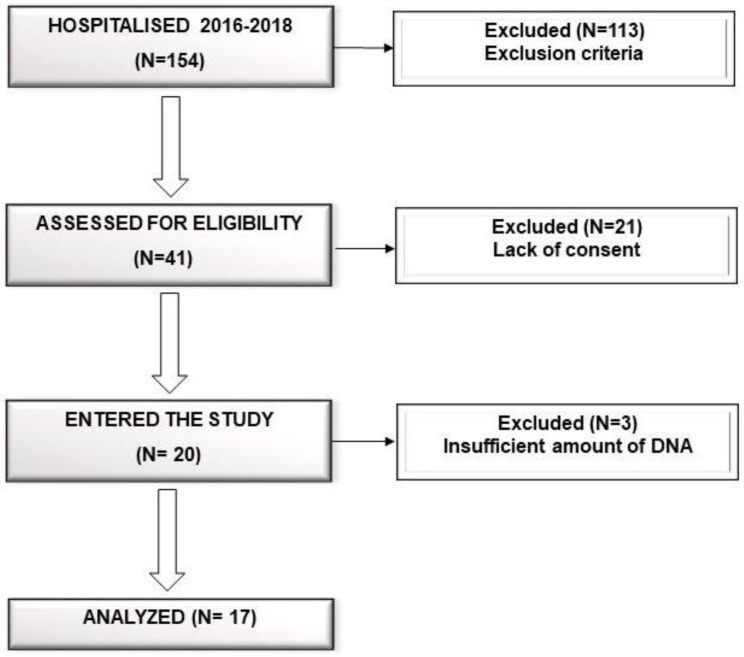
Study flow chart.

The study protocol was approved by the Bioethics Committee of the Pomeranian Medical University in Szczecin. All participants received a written description of the study aims, and gave written informed consent prior to participation. Participants were recruited as inpatients via the Department of Psychiatry in Szczecin between October 2016 and May 2018, during which, only 20 psychiatric inpatients met the inclusion criteria (Flow chart in Figure 1). They were diagnosed with major depressive disorder—depressive episode of at least moderate severity according to the ICD-10 criteria (F32.1, F32.2, F33.1, F33.2). The inclusion criteria were:

- Aged between 18–65 years;
- Depressive episode of at least moderate severity according to ICD-10;
- Written informed consent of participation.
- Exclusion criteria were as follows:
- Total or partial incapacitation;
- Comorbidity with severe depressive episode and psychotic symptoms (F32.3), schizophrenia, schizotypal or delusional disorders (F20–F29), bipolar disorder (F31), mental retardation (F70–F79), active addiction to alcohol or other psychoactive substances (F10–F19) excluding nicotine (F17) and caffeine (F15), organic—including symptomatic—mental disorders (F00–F09), epilepsy (G40);
- Inflammatory and infectious diseases;
- Poor general health, including cancer, gastrointestinal diseases (tumors, inflammations, celiac disease, food intolerance, colitis ulcerosa, and Crohn’s Disease);
- Diabetes and thyroid dysregulation;
- Treatment with antibiotics, non-steroidal anti-inflammatory drugs, probiotics, corticosteroids, immunosuppressive and immunomodulating drugs, or proton pump inhibitors for at least 3 months prior to baseline assessment;
- A history of treatment-resistant depression.

The study was conducted in a psychiatric ward setting. Consequently, all participants were subjected to the same daily routine, including physical exercise (daily morning exercise and a walk with a therapist), occupational therapy, and psycho-educational activities. Two senior psychiatrists performed the psychiatric and basic physical examinations, whereas, a gastroenterologist performed a comprehensive physical examination.

Patients received a standard hospital diet, balanced by a hospital dietician, in accordance with the Polish standards for hospitalized patients, i.e., the same diet with mean values of 2995 ± 93 kcal, 106 ± 14 g of total protein, 420 ± 24 g of carbohydrates, and 102 ± 10 g fat per day. Detailed nutritional data on the diet during hospitalization, including fiber consumption, are presented in Table 1.

**Figure 2:**
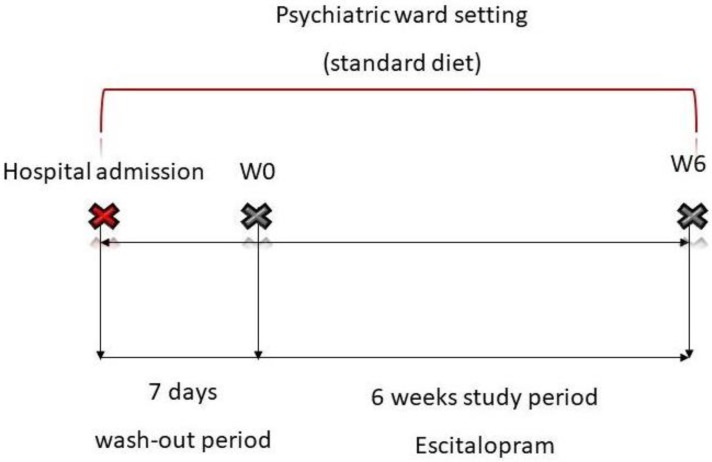
Study schema.

A total of 17 patients were included in the study (eight males and nine females). Three individuals were excluded from the final analysis, due to the insufficient DNA amount isolated from the stool samples. All included patients underwent at least a 7-day washout from all medications. The washout was conducted in the psychiatric ward setting, during this period patients underwent the same daily hospital routine and received a standard hospital diet, as was previously described. After washout the first feces sample was collected (week 0; W0), followed by escitalopram administration at an initial dose of 5 mg per day. Further drug-dosing used for downstream therapy was individually adjusted (5–20 mg per day), depending on the severity of depressive symptoms and the patient’s clinical condition. A second stool sample was collected after 6 weeks (W6). The study schema is presented in Figure 2. Fecal samples were collected using microbiome sampling kits purchased from uBiome (https://ubiome.com). DNA extraction and sequencing of the V4 hypervariable regions of the bacterial 16S rRNA gene was conducted by uBiome, according to an in-house protocol using the Illumina NextSeq 500 platform in a 150 bp paired-end modality with the following primers (515F: GTGCCAGCMGCCGCGGTAA and 806R: GGACTACHVGGGTWTCTAAT) [39]. For further analysis, we used taxonomy annotation tables stored in JSON-formatted files provided by uBiome. Each table contained sample ID, taxon name according to NCBI, taxonomic rank of the taxon, absolute (counts) and relative (percent) number of reads mapped to the taxon.

### 2.2. Processing of Raw Data and Statistical Analysis

A downstream analysis was performed using the R software (version 3.5.1, https://cran.r-project.org/) and *Phyloseq* package (version 1.24.2), for the handling and analysis of high-throughput microbiome data [40]. First, the JSON taxonomy-formatted files containing taxonomy annotation tables were downloaded from uBiome, and used to create the *Phyloseq* object using the *phyloseq_from_JSON* function from the *Actino* package (Phyloseq utilities for personal microbiome, version 0.1.12, available online: https://github.com/richardsprague/actino, accessed on 7 October 2018), and a mapping file containing sample metadata, such as sample ID, patient ID, and patients’ demographic and clinical characteristics. As a result, a phyloseq-class experiment-level object was created consisting of an operational taxonomy unit (OTU) table (526 taxa and 34 samples), a taxonomy table (526 taxa by 7 taxonomic ranks), and sample meta-data.

The gut microbial alpha diversity was estimated based on the originally observed count values prior to any pre-processing, which was evaluated using the Chao1 index as a measure of species richness. The Shannon and inverse Simpson (invSimpson) indices were used as measures of taxa richness and evenness [41]. A paired Wilcoxon signed-rank test (two-sided) was used to test whether species richness, in addition to the Shannon and inverse Simpson diversities, were significantly different between paired samples at two consecutive time points (W0 and W6). Prior to the beta diversity analysis, an unsupervised data filtering was conducted using the prevalence of taxa defined as the proportion of samples in which the taxa appeared at least once. Two hundred twenty-nine taxa were filtered out, leaving 297 taxa with prevalence greater than 5% (e.g., taxa that appeared in at least two samples, 5.9%). A proportional transformation of abundances was implemented using the median sequencing depth as a scaling factor. Only the five most abundant phyla were kept in the next step (*Firmicutes*, *Bacteroidetes*, *Actinobacteria*, *Proteobacteria*, *Verrucomicrobia*), which reduced the number of OTU from 297 to 293. Beta diversity was analyzed using principal coordinate analysis (PCoA) on Bray–Curtis distance matrices generated from the normalized OTU tables. Any outliers identified when plotting the ordination diagrams were removed before further analysis. To analyze the compositional changes in bacterial communities of patients at two time points (W0 and W6), a permutation multivariate analysis of variance (PERMANOVA) implemented in the PERMANOVA+ add-on package of PRIMER 7 (version 7.0.13) was used.

The abundance of various bacterial taxa, at different taxonomic levels, in patients at two time points (W0 and W6) were compared using a paired *t*-test. The min P step-down adjusted *p* values (FWER), and the Benjamini–Hochberg method was used to control the False Discovery Rate (FDR).

## 3. Results

Stool samples were collected from 17 patients (eight males and nine females, aged 21.0–64.0 years, mean ± SD 42.5 ± 13.9 years). Patient clinical characteristics are presented in Table 2.

**Figure 3:**
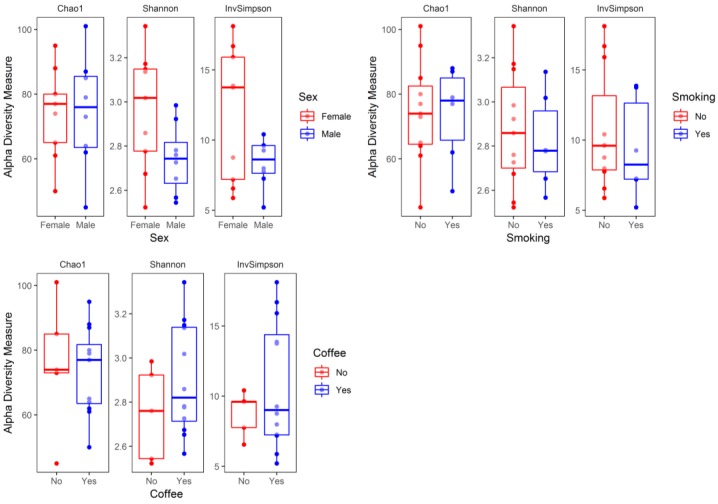
Alpha diversity metrics—Chao1, Shannon, and inverse Simpson (invSimpson) with respect to sample metadata (sex, smoking status, and coffee consumption) at baseline (W0). The boxplots represent diversity measures (center line—median, lower, and upper hinges correspond to the first (Q1) and third (Q3) quartiles; whiskers—the upper whisker is located at the smaller of the maximum alpha diversity measures and Q3 + 1.5 × IQR (Q3 − Q1); the lower whisker is located at the larger of the minimum alpha diversity measures and Q1 − 1.5 × IQR).

**Figure 4:**
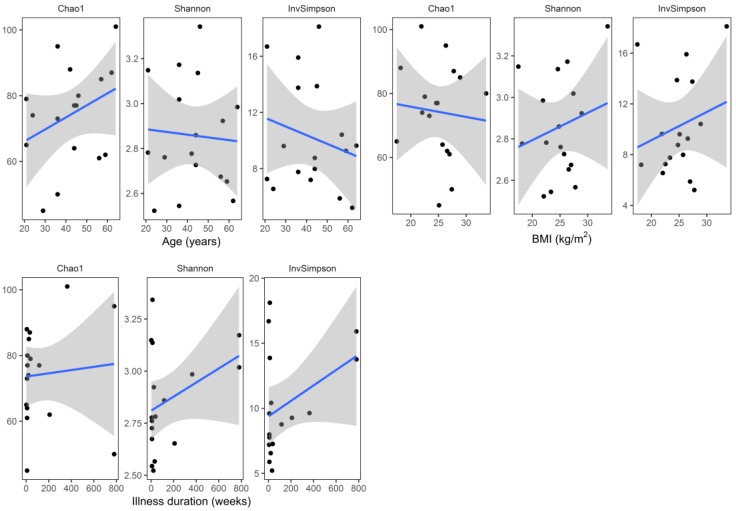
Correlations (scatter plots) between alpha diversity metrics—Chao1, Shannon, and inverse Simpson (invSimpson), and sample metadata (age, BMI, and illness duration) at baseline (W0). The regression lines (colored blue) were fitted using the linear model; grey shading areas represent confidence bounds.

Median (Q1–Q3) number of reads in the samples at baseline (W0) and at the end of hospitalization (W6) were 48,194 (36,407–71,405) and 36,440 (26,575–61,171), respectively. Baseline (W0) alpha diversity measures (Chao1, Shannon, and inverse Simpson) did not differ significantly (two sided Mann–Whitney test) with respect to sex (*p* = 1.0, *p* = 0.092, *p* = 0.312, respectively), smoking status (*p* = 0.920, *p* = 0.802, *p* = 0.451, respectively), and coffee consumption (*p* = 0.958, *p* = 0.225, *p* = 0.792, respectively) (Figure 3). Similarly, there was no significant correlation (Spearman coefficient, and two-sided test) between baseline alpha diversity metrics (Chao1, Shannon, inverse Simpson) and age (R = 0.31, *p* = 0.223; R = −0.05, *p* = 0.859; R = −0.07, *p* = 0.793, respectively), BMI (R = −0.11, *p* = 0.663; R = 0.08, *p* = 0.758; R = 0.13, *p* = 0.626, respectively), and illness duration (R = 0.28, *p* = 0.283; R = 0.20, *p* = 0.442; R = 0.17, *p* = 0.526, respectively) (Figure 4).

**Figure 5:**
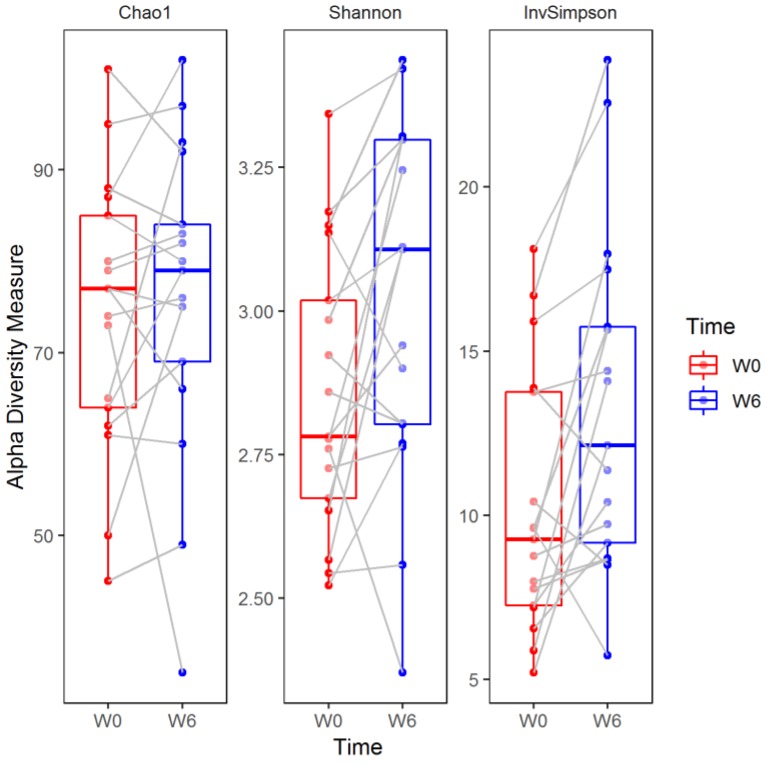
Alpha diversity measures in patients at baseline (W0) and after six weeks of hospitalization (W6). The boxplots represent the diversity measures (center line—median, lower, and upper hinges correspond to the first (Q1) and third (Q3) quartiles; whiskers—the upper whisker is located at the smaller of the maximum alpha diversity measures and Q3 + 1.5 × IQR (Q3 − Q1), the lower whisker is located at the larger of the minimum alpha diversity measures and Q1 − 1.5 × IQR). Paired samples are connected by grey lines. W0 and W6 represent time points.

The Chao1 index did not change significantly over the six weeks of observation (median (Q1–Q3), W0: 77 (64–85) versus W6: 79 (69–84), two sided Wilcoxon signed-rank test, False Discovery Rate-adjusted *p* (*q*) = 0.449). In contrast, the Shannon (W0: 2.78 (2.67–3.02) versus W6: 3.11 (2.80–3.30)) and inverse Simpson (W0: 9.26 (7.26–13.76) versus W6: 12.13 (9.17–15.73)) indices increased significantly compared to baseline values (*q* = 0.031 and *q* = 0.011, respectively; Figure 5).

**Figure 6:**
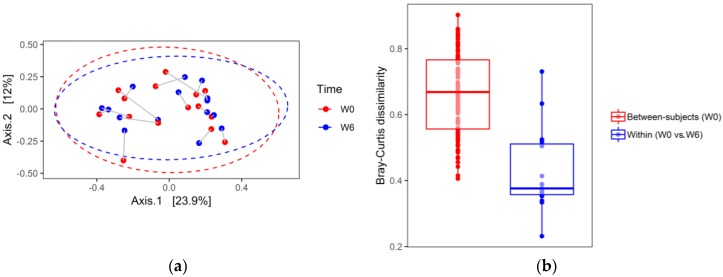
Gut microbial composition changes during six weeks of hospitalization in patients with a depressive episode, treated with escitalopram. (**a**) Principal coordinate analysis based on Bray–Curtis dissimilarities calculated using normalized count data, median sequencing depth, and the five most abundant phyla. Two outlier samples were removed (one male and one female), *n* = 15. Samples are colored according to time points. Grey lines connect projections of samples from the same patients. Ellipses correspond to 95% confidence intervals for two time points (W0 and W6) assuming a multivariate normal distribution. (**b**) The boxplot shows Bray–Curtis dissimilarities (center line—median, lower, and upper hinges correspond to the first (Q1) and third (Q3) quartiles; whiskers—the upper whisker is located at the smaller of the maximum Bray–Curtis measures and Q3 + 1.5 × IQR (Q3 − Q1); the lower whisker is located at the larger of the minimum Bray–Curtis measures and Q1 − 1.5 × IQR). W0 and W6 represent time points.

The principal coordinate analysis (PCoA) based on Bray–Curtis dissimilarities is shown in Figure 6a,b (a first pass analysis demonstrated two outliers that have been removed). In contrast to alpha diversity analysis, PCoA did not show substantial differences in stool microbial compositions between W6 and W0, indicated by a large overlap between ellipses. To test differences in the microbial community at the two time points (W0 and W6), the repeated measures permutation analysis of variance (PERMANOVA) was applied to the Bray–Curtis dissimilarities. PERMANOVA with 9999 permutations of residuals under a reduced model did not reveal significant compositional changes in the structure of the bacterial community between W0 and W6 (Pseudo-F (1, 14) = 0.561, *p* = 0.842). Furthermore, stool samples collected from the same patient at W0 and W6, projected to a 2D space analysis, were located close to each other. These data suggest that intra-individual diversity in the gut’s bacterial community is smaller than inter-individual diversity. In line with this observation, between-subject W0 Bray–Curtis dissimilarities were significantly higher than W0–W6 within-subject (same donor) differences (median (Q1–Q3): 0.68 (0.56–0.77) vs. 0.38 (0.35–0.52), two sided Mann–Whitney test *p* < 0.00001; Figure 6).

**Figure 7:**
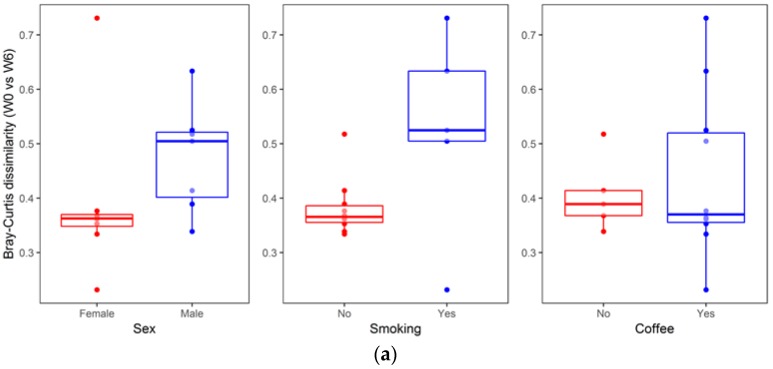

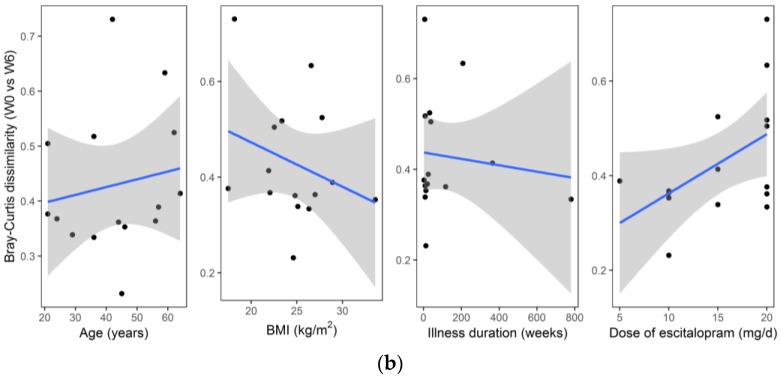
Same-donor Bray–Curtis dissimilarities (W0 vs. W6) association with sample metadata (age, sex, BMI, illness duration, smoking status, and coffee consumption). (**a**) The boxplots represent the within-subject (same donor) beta diversity measures, Bray–Curtis dissimilarities (center line—median, lower, and upper hinges correspond to the first (Q1) and third (Q3) quartiles; whiskers—the upper whisker is located at the smaller of the maximum beta diversity measures and Q3 + 1.5 × IQR (Q3 − Q1); the lower whisker is located at the larger of the minimum beta diversity measures and Q1 − 1.5 × IQR). (**b**) Correlations (scatter plots) between same-donor Bray–Curtis dissimilarities and sample metadata (age, BMI, illness duration and dose of escitalopram). The regression lines (colored blue) were fitted using the linear model; grey shading areas represent confidence bounds.

An analysis of the association between sample metadata and same-donor Bray–Curtis dissimilarities (W0 vs. W6) is summarized in Figure 7. No significant differences were detected with respect to sex (median (Q1–Q3): Females, 0.36 (0.34–0.37) vs. males, 0.50 (0.39–0.52), two sided Mann–Whitney *p* = 0.073), smoking status (no smoking, 0.37 (0.35–0.39) vs. smoking, 0.52 (0.50–0.63), *p* = 0.098), and coffee consumption (no coffee, 0.39 (0.37–0.41) vs. coffee, 0.37 (0.35–0.52), *p* = 0.951). Likewise, no significant correlation was found for same-donor Bray–Curtis dissimilarities with respect to age (R = 0.22, *p* = 0.438), BMI (R = −0.22, *p* = 0.428), dose of escitalopram (R = 0.37, *p* = 0.169) or illness duration (R = −0.02, *p* = 0.944).

Differential abundance testing revealed no significant differences between the two time points (W0 vs. W6) measured at different taxa levels, when multiple hypothesis testing procedures were implemented (Table 3, Table 4, Table 5 and Table 6). Table 3, Table 4, Table 5 and Table 6 summarize the results of comparing bacterial communities at the level of phylum (Table 3), class (Table 4), order (Table 5), and family (Table 6).

## 4. Discussion

To the best of our knowledge, this is the first study analyzing fecal microbiota composition in human patients subjected to hospitalization secondary to a depressive episode. We found a significant increase in fecal microbiota biodiversity, mainly alpha diversity; whereas taxa were not differentially abundant after six weeks of hospitalization, and concomitant therapy using 5–20 mg of escitalopram. Alpha diversity indices at baseline (W0) were not associated with patient demographics and clinical characteristics (Figure 3 and Figure 4). Although no changes in gut microbiota richness (Chao1 index) were demonstrated, the other two alpha diversity metrics (Shannon and inverse Simpson) increased significantly compared to W0 (Figure 5). Shannon and Simpson diversity indices reflect both species richness and evenness, yet the former is more sensitive to species richness, whereas the latter is biased toward species evenness [42].

Further analyses of microbiome composition changes during hospitalization (ordination and differential abundance testing) have shown (Figure 6), that between-subjects Bray–Curtis dissimilarities at baseline were significantly higher than same donor (within-subject) dissimilarities (W0 vs. W6). It means that individual microbiome stability was not affected by hospitalization.

While studying the effects of antidepressant drugs on gut microbiota, Cussotto et al. [37] administered three different SSRIs, including escitalopram, to rats, they did not however observe any significant changes in bacterial community structure as measured by alpha diversity. Although Cussotto et al. found a significant difference in species variability and composition between rats treated with escitalopram and those receiving vehicle, the principal coordinate analysis of Bray–Curtis distances showed that the groups largely overlapped. Recent studies on the influence of increased alpha diversity on the CNS reported conflicting results. For example, Jiang et al. observed increased alpha diversity in patients with active MDD [18]. While Naseribafrouei et al. [43] found no significant difference in alpha diversity when comparing fecal microbiota of depressive and healthy individuals. Others also reported diminished richness and diversity of fecal bacteria in depressive persons [19,20]. Painold et al. [21] observed a negative correlation between microbial alpha diversity and illness duration, in patients with depressive episodes suffering from bipolar disorder (BD). Additionally, Kleiman et al. [17] reported that bacterial diversity decreased with elevated depression intensity in acute Anorexia Nervosa. In this context, we believe that ambiguity must be eliminated when determining if increased alpha diversity has a positive effect on the course of depression or not. The relationship between alpha diversity and clinical effects during hospitalization were not assessed for this study’s participants, however, all patients achieved clinical improvement. Thus, it could be hypothesized that increased diversity may have a beneficial effect on patients’ mental health. It seems to be in line with the latest report by Huang et al. [44] who reported that the alpha diversity in MDD patients was lowered in comparison to healthy controls with Firmicutes phylum being the most decreased one. Nevertheless, the hypothesis that a more diverse fecal microbial community can positively influence patients’ health [45] was beyond the scope of this study, and awaits validation in future proof of concept (PoC) studies. Of note and according to Shade, diversity is neither good nor bad, it simply “is” [46]. Therefore, it must be stressed that increased alpha diversity is not always beneficial for health.

Hospitalization per se can also influence the fecal microbiota composition, no evidence of such data was found for depressive patients. However, Kleiman et al. [17] reported that during hospitalization, patients with anorexia nervosa (AN) exhibited significant changes in taxa abundance and beta (between-sample) diversity. Of course, AN patients differ from our population (nutritional problems are crucial in AN), but these data indicate that changes to diet and life style during hospitalization affect gut microbiota. Interestingly, well known factors that strongly influence alpha diversity are food and dietary habits [47], however, evidence of their nervous system-related effects remains scarce. It is likely that a regular and diverse diet at the time of hospitalization positively affects alpha diversity levels, considering that patients ingested more than 30 g of fiber per day during their hospital stay. There seems to be a higher-than-normal amount of fiber in the diet of depressive patients. Our recently published data show that the mean consumption of fiber among Polish depressive women was 19.01 ± 11.09 g/day (median: 17.39; Min: 1.85; Max: 60.89 g/day) [48]. Fiber is a microbiota energy source, and a substrate for short chain fatty acid synthesis. It was recently demonstrated [49] that the growth of beneficial bacteria within the gut microbiota is favorably affected by fermentable fiber ingestion. Schnorr et al. found increased microbial richness and biodiversity in the Hadza hunter-gatherer individuals (whose diet is rich in fiber) when compared to urban Italian controls [50]. However, it was not proved that increased richness in this population was caused by consumption of fiber. On the other hand, a meta-analysis—including 64 studies comprising 2099 participants—by So et al. reported that dietary fiber intake is associated with higher fecal abundance of *Bifidobacterium* and *Lactobacillus* spp., but does not influence α-diversity [51]. In our study however, we did not monitor nutrition intake prior to hospital admission. Altogether, the impact of diet on microbiota composition during a depressive episode requires clarification.

Beyond diet, other environmental factors—smoking status and physical activity—could affect the composition of gut microbiota. However, the analysis of within-subject Bray–Curtis dissimilarities with respect to patients’ metadata revealed no significant associations (there was a statistical trend for sex and smoking status, Figure 7).

Despite the positive changes in alpha diversity at the end of the study, we failed to find a differential abundance of OTUs during the hospital stay. In vitro and in vivo studies demonstrate escitalopram’s antibacterial properties against pathogenic bacteria, such as *Staphylococcus aureus*, *Pseudomonas aeruginosa*, *Klebsiella pneumoniae*, and *Proteus mirabilis Enterobacter cloacae* [36], or its ability to inhibit the growth of *Escherichia coli* APC105 [37]. Our study did not, however, confirm the effects of this antidepressant on fecal bacterial abundance. It is noteworthy that in contrast to alpha diversity; where the bacterial composition is summarized by a single measure (i.e., Shannon index) and subsequently compared between groups, testing differential abundance depends on the comparison of each taxon separately. Similarly, Cussotto et al. did not observe the effect of escitalopram on the growth of *Lactobacillus rhamnosus* in vitro, or the composition of other bacterial species in rats [37].

### Limitations

An important limitation of our study was a small cohort size. Due to high inter individual variation observed in our study, as well as a reasonably stable microbiome, a higher number of patients could have possibly led to significant differences of bacterial abundance being found in the beginning and at the end of hospitalization. The main reasons for the low number of patients enrolled were the adherence to very rigorous inclusion and exclusion criteria, lack of consent, and the limited time frame of the study—17 months.

In our study, patients’ results were not compared to a healthy control group or subjects receiving placebo dosing. Such comparisons would require the control group’s dietary patterns and lifestyle choices to be similar to those of the patients’. However, both the diet and general lifestyle characteristics of patients with depression were found differ significantly from those of healthy individuals [52]. Furthermore, our study took place in a public hospital, deeming placebo use debatable from ethical and organizational points of view. Our study design has important strengths, such as comparable conditions as far as the diet, drug intake, and clinical monitoring. Nonetheless, some limitations exist, including the change of dietary and living conditions, due to hospitalization.

Another limitation of this study is the lack of analysis of the metabolic function of microbiota, or the association between the composition of microbiota and the clinical course of illness, including inflammatory markers defining the function of the intestinal barrier. Furthermore, our research is solely based on stool analysis, hence, we cannot exclude conflict resulting from the analysis of microbiota associated with mucous membranes. However, conducting such studies in psychiatric patients would be difficult for ethical reasons, where the availability of endoscopic biopsies would require invasive examinations. Performing such procedures on patients without gastrointestinal complaints would be of significant limitation. Due to the intensely growing interest from the psychiatric community in psychotropic drugs problems and its microbiota-related effects, we believe that our results will not only expand on knowledge within this field, but will also serve as a platform for further research.

## 5. Conclusions

In this study, we demonstrated that a six-week-long hospital stay, due to an acute depressive episode increased biodiversity, mainly alpha diversity, of fecal microbiota, although no difference in taxa abundance was found at different study time points. We did not prove that increased alpha biodiversity of fecal microbiota had a positive impact on patients’ mental health. We have also found that individual microbiome stability was not affected by hospitalization. Further studies, including analysis of the metabolic function of microbiota and function of the intestinal barrier, as well as the associations between the composition of microbiota and the clinical course of illness, are necessary to fully understand the influence of hospitalization (including escitalopram treatment) on gut microbiota and its clinical significance.

## Figures and Tables

**Table 1:** Average daily macronutrient intake during hospital stay.

Variable	Recommended Daily Intake *	Result	
		Mean	Range
Energy, kcal		2995	2865–3165
Protein, g		106	89–122
Fat, g		102	91–116
Carbohydrates, g		420	387–442
Fiber, g	>25	31	28–33
% Energy from protein	10–20	14	12–15
% Energy from fat	20–35	31	29–33
% Energy from carbohydrates	45–65	56	54–56

* For healthy Polish people [38].

**Table 2:** Clinical characteristics of patients included in the study.

Characteristic	*n* = 17
Age, years	42.5 ± 13.9 (21.0–64.0)
Sex (M/F), *n* (%)	8/9 (47/53)
BMI, kg/m^2^	24.9 ± 3.9 (17.5–33.6)
Smoking (Y/N), *n* (%)	6/11 (35/65)
Coffee (Y/N), *n* (%)	12/5 (71/29)
Age of diagnosis of depression, years	40.1 ± 14.6 (21.0–62.0)
Illness duration, weeks	143 ± 258 (2–780)
Age of onset of psychiatric treatment, years	38.6 ± 14.4 (19.0–62.0)
Current episode duration, weeks	11.5 ± 7.9 (2.0–32.0)

Mean ± standard deviation (range).

**Table 3:** Comparison of taxon abundance between the two time points (W0 and W6) at the level of phylum.

Phylum	W0 (%)	W6 (%)	Raw *p* ^†^	*p* ^1^	*p* ^2^
*Firmicutes*	46.9	42.7	0.526	0.792	0.658
*Bacteroidetes*	40.0	47.4	0.239	0.667	0.650
*Actinobacteria*	5.5	4.6	0.661	0.792	0.661
*Proteobacteria*	3.5	4.2	0.390	0.792	0.650
*Verrucomicrobia*	4.1	1.0	0.348	0.792	0.650

^1^ permutation adjusted *p* value using the step-down min P procedure to control for family-wise type I error; ^2^ FDR-adjusted *p* value for the Benjamini–Hochberg method to control for False Discovery Rate; ^†^ paired *t*-test.

**Table 4:** Comparison of taxon abundance between the two time points (W0 and W6) at the level of class.

Class	W0 (%)	W6 (%)	Raw *p* ^†^	*p* ^1^	*p* ^2^
*Bacteroidia*	40.0	47.4	0.239	0.877	0.902
*Clostridia*	43.0	39.7	0.620	0.996	0.902
*Actinobacteria*	5.5	4.6	0.661	0.996	0.902
*Verrucomicrobiae*	4.1	1.0	0.348	0.952	0.902
*Negativicutes*	2.4	2.3	0.902	0.998	0.902
*Betaproteobacteria*	1.6	2.1	0.393	0.963	0.902
*Gammaproteobacteria*	1.3	1.4	0.852	0.998	0.902
***Bacilli***	**1.2**	**0.3**	**0.009**	**0.076**	**0.085**
*Deltaproteobacteria*	0.7	0.8	0.610	0.996	0.902
*Erysipelotrychia*	0.3	0.4	0.893	0.998	0.902

^1^ permutation adjusted *p* value using step-down min P procedure to control for family-wise type I error; ^2^ FDR-adjusted *p* value for the Benjamini–Hochberg method to control for False Discovery Rate; ^†^ paired *t* test. Statistically significant results were highlighted by bold.

**Table 5:** Comparison of taxon abundance between the two time points (W0 and W6) at the level of order.

Order	W0 (%)	W6 (%)	Raw *p* ^†^	*p* ^1^	*p* ^2^
*Bacteroidales*	40.0	47.4	0.239	0.954	0.998
*Clostridiales*	43.0	39.7	0.620	0.999	0.998
***Lactobacillales***	**1.2**	**0.3**	**0.009**	**0.096**	**0.119**
*Selenomonadales*	2.4	2.3	0.902	0.999	0.998
*Bifidobacteriales*	3.7	2.7	0.594	0.999	0.998
*Coriobacteriales*	1.8	1.9	0.700	0.999	0.998
*Actinomycetales*	0.02	0.02	0.385	0.992	0.998
*Enterobacteriales*	1.2	1.2	0.927	0.999	0.998
*Erysipelotrichales*	0.3	0.4	0.893	0.999	0.998
*Burkholderiales*	1.6	2.1	0.393	0.992	0.998
*Desulfovibrionales*	0.7	0.8	0.610	0.999	0.998
*Pasteurellales*	0.1	0.1	0.844	0.999	0.998
*Neisseriales*	0.0006	0.0008	1.0	1.0	1.0
*Verrucomicrobiales*	4.1	1.0	0.348	0.988	0.998

^1^ permutation adjusted *p* value using step-down min P procedure to control for family-wise type I error; ^2^ FDR-adjusted *p* value for the Benjamini–Hochberg method to control for False Discovery Rate; ^†^ paired *t* test. Statically significant results were highlighted by bold.

**Table 6:** Comparison of taxon abundance between the two time points (W0 and W6) at the level of family.

Family	W0 (%)	W6 (%)	Raw *p* ^†^	*p* ^1^	*p* ^2^
*Bacteroidaceae*	29.9	33.4	0.484	1.0	0.987
*Lachnospiraceae*	26.9	24.6	0.617	1.0	0.987
*Streptococcaceae*	0.6	0.3	0.191	0.992	0.987
*Lactobacillaceae*	0.4	0.06	0.110	0.934	0.987
*Porphyromonadaceae*	6.7	8.7	0.160	0.986	0.987
*Bifidobacteriaceae*	3.7	2.7	0.594	1.0	0.987
*Veillonellaceae*	0.9	0.7	0.694	1.0	0.987
*Coriobacteriaceae*	1.7	1.9	0.700	1.0	0.987
***Rikenellaceae***	**2.8**	**4.8**	**0.015**	**0.282**	**0.508**
*Actinomycetaceae*	0.02	0.02	0.423	1.0	0.987
*Ruminococcaceae*	15.5	14.4	0.753	1.0	0.987
*Erysipelotrichaceae*	0.3	0.4	0.893	1.0	0.987
*Enterobacteriaceae*	1.2	1.2	0.927	1.0	0.987
*Prevotellaceae*	0.5	0.5	0.888	1.0	0.987
*Acidaminococcaceae*	1.5	1.6	0.907	1.0	0.987
*Sutterellaceae*	1.6	2.0	0.403	1.0	0.987
*Desulfovibrionaceae*	0.7	0.8	0.610	1.0	0.987
*Clostridiales Family XI. IncertaeSedis*	0.001	0.001	0.645	1.0	0.987
*Peptostreptococcaceae*	0.3	0.5	0.504	1.0	0.987
*Enterococcaceae*	0.2	0.01	0.252	0.999	0.987
*Pasteurellaceae*	0.1	0.1	0.844	1.0	0.987
*Eubacteriaceae*	0.007	0.002	0.083	0.869	0.987
*Clostridiaceae*	0.005	0.004	0.922	1.0	0.987
*Verrucomicrobiaceae*	4.1	1.0	0.348	1.0	0.987
*Oxalobacteraceae*	0.02	0.03	0.449	1.0	0.987
*Oscillospiraceae*	0.0003	0.002	0.499	1.0	0.987
*Neisseriaceae*	0.001	0.001	1.0	1.0	1.0
*Leuconostocaceae*	0.001	0.002	0.879	1.0	0.987
*Corynebacteriaceae*	0.001	0.001	0.506	1.0	0.987
*Christensenellaceae*	0.0006	0.002	0.503	1.0	0.987
*Catabacteriaceae*	0.0003	0.0002	1.0	1.0	1.0
*Carnobacteriaceae*	0.003	0.001	0.254	0.999	0.987

^1^ permutation adjusted *p* value using step-down min P procedure to control for family-wise type I error; ^2^ FDR-adjusted *p* value for the Benjamini–Hochberg method to control for False Discovery Rate; ^†^ paired *t* test. Statically significant results were highlighted by bold.
